# A Case of “Refractory” Neuropsychiatric Lupus Responsive to Anticoagulation

**DOI:** 10.1155/2017/5726180

**Published:** 2017-01-30

**Authors:** Rui Wu, Sun Hu

**Affiliations:** Department of Rheumatology, The First Affiliated Hospital of Nanchang University, Nanchang, China

## Abstract

Neuropsychiatric disorder is a severe complication in 14% to 75% of systemic lupus erythematosus (SLE) patients, which can result in significant morbidity. A 15-year-old female SLE patient with coexistence of dural sinus thrombosis and intracerebral hemorrhage resistant to two pulses of high dose of glucocorticoid was treated with anticoagulation of the low-molecular-weight [LMW] heparin subcutaneously followed by warfarin. The patient demonstrated a remarkable clinical response.

## 1. Introduction

Systemic lupus erythematosus (SLE) is a chronic, autoimmune disease with multiple-systemic disorders. Involvement of the nervous system has been recognized ever since the disease was first reported, which may involve both the central and peripheral nervous systems. In previous studies, neuropsychiatric disorder was reported in 14% to 75% of SLE patients [1]. Although there have been significant advances in understanding of some aspects of neuropsychiatric SLE in recent years, management of nervous system disease continues to be a challenging problem that can result in significant morbidity. To treat many NPSLE manifestations, immunosuppressive therapy with high-dose corticosteroids and cyclophosphamide has been commonly used according to the individual patient's needs [2]. We report a rare case of NPSLE with coexistence of dural sinus thrombosis and intracerebral hemorrhage that had an inadequate response to high-dose corticosteroids and cyclophosphamide but responded dramatically to anticoagulation.

## 2. Case Report

**Figure 1:**
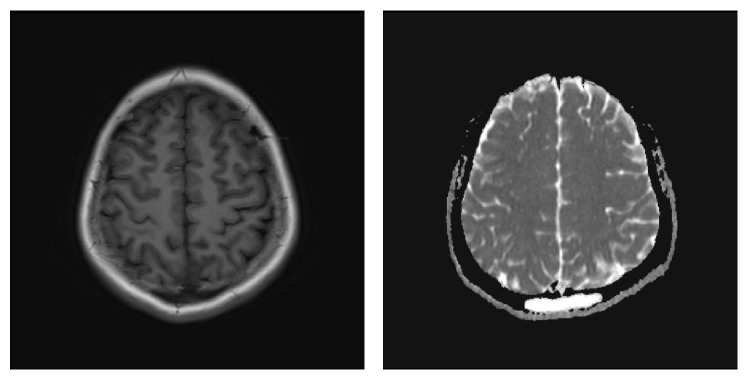
Abnormal signal of the occipital scalp.

**Figure 2:**
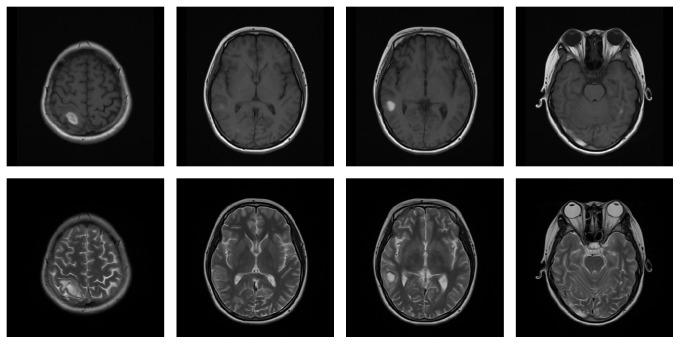
Hemorrhage of left temporal lobe, right parietal lobe, temporal lobe, occipital lobe, and right cerebellar hemisphere and suspicious thrombosis of sagittal sinus and right transverse sinus.

**Figure 3:**
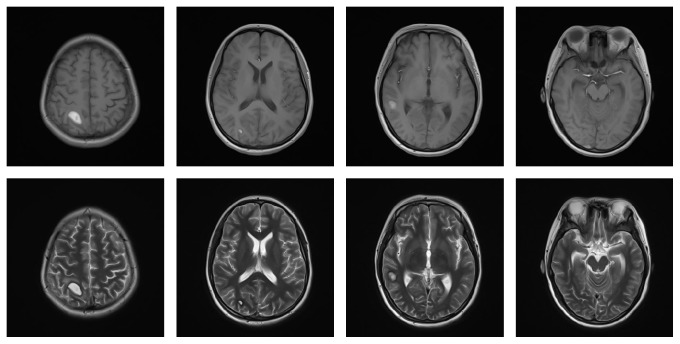
Brain MRI improved after one week of anticoagulation.

**Figure 4:**
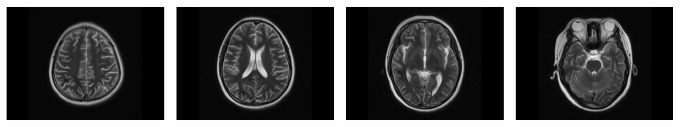
Approximately 6 weeks after initial treatment with anticoagulation.

A 15-year-old female was admitted to our hospital for fever, dizziness, trouble speaking, and blurred vision for two weeks. She initially presented with fever, dizziness, and cough. Serologies were significant for decreased blood platelet of 40 × 10^9^/L and decreased complement level, a positive ANA of 1 : 100, positive anti-Rib-p, anti-SSA and positive Coomb's test. Brain magnetic resonance imaging (MRI) showed abnormal signal of the occipital scalp (Figure 1). Cerebrospinal fluid was normal. Since her diagnosis of SLE approximately one week prior to the admission, she had been treated with pulse methylprednisolone (MP) therapy (500 mg intravenous [i.v.] daily for 3 days) and iv human polyvalent immunoglobulin pulses (IVIg) in a dose of 20 g once daily for 5 days followed with 80 mg of MP and iv cyclophosphamide for presumed CNS involvement. She was admitted for developing trouble speaking, blurred vision, and headache. At the time of admission, she had drowsiness, confusion, difficulty concentrating, speech disorder, and double vision. Complete blood count showed white blood cell 8.49 × 10^9^ and red blood cell 2.95 × 10^12^/L and platelet count was 40 × 10^9^/L. Activated partial thromboplastin time (APTT) was increased (89.7 s) which suggested positive Lupus anticoagulant (LA). Anticardiolipin antibody of IgG isotype and anti-b2 glycoprotein I antibodies of IgG and IgM isotypes were positive. A diagnosis of neuropsychiatric lupus and secondary antiphospholipid syndrome was made. The second pulse methylprednisolone (MP) therapy (500 mg intravenous [i.v.] daily for 3 days) was given one week after the patient was admitted to hospital for worsening of neurologic signs and symptom. Despite two-pulse MP therapies and intravenous cyclophosphamide, the patients continued to develop new nervous system symptoms such as dysphoria, illusion, somnolence, and bilateral papillary edema. She was remained on 80 mg of MP and treated with antipsychotic drug of Seroquel and Mannitol to reduce intracranial pressure. The brain MRI was ordered again and suggested hemorrhage of left temporal lobe, right parietal lobe, temporal lobe, occipital lobe, and right cerebellar hemisphere and suspicious thrombosis of sagittal sinus and right transverse sinus (Figure 2). Due to positive ACL and LA, a decision was made to start treatment with anticoagulation of the low-molecular-weight [LMW] heparin subcutaneously followed by warfarin with target INR 2–2.5 and aspirin for clinical diagnosis of cerebral venous sinus thrombosis (CVST). One week after the treatment of anticoagulation brain MRI showed subacute hemorrhage of left temporal lobe, right parietal lobe, temporal lobe, occipital lobe, and right cerebellar hemisphere and subacute thrombosis of the right transverse sinus (Figure 3). Two weeks later, the patient had significant improvement in headache and consciousness. Warfarin and aspirin were continued and MP remained at 40 mg once daily. Approximately 6 weeks after initial treatment with anticoagulation, the patient had complete resolution of neurologic symptoms and brain MRI (Figure 4). It demonstrated that noninflammatory microangiopathy and thrombosis were the predominant pathogenetic pathway in the patient's neuropsychiatric disorder.

## 3. Discussion

In the American College of Rheumatology (ACR) classification criteria [3], NP manifestations of SLE include two features, namely, seizures and psychosis. But it is currently known that NPSLE is a much broader spectrum of disease, which may involve both the central and peripheral nervous systems. Central nervous system (CNS) involvement predominates over peripheral nervous system disease and may take the form of diffuse disease (e.g., psychosis and depression) or focal disease (e.g., cerebrovascular disease and transverse myelitis) depending upon the anatomic location of pathology. Cerebrovascular disease is severe complication of NPSLE which included cerebral infarction, intracerebral hemorrhage, and subarachnoid hemorrhage. It is reported to occur in 0.4–0.7% of SLE patients [4]. Although vasculitis is basic pathological changes in most systemic involvements [5], noninflammatory microangiopathy is rather more frequent instead of vasculitis in CNS disorder. Venous sinus thrombosis is one of noninflammatory manifestations of NPSLE and more common in patients with antiphospholipid antibodies (aPL), such as anticardiolipin antibodies (aCL), lupus anticoagulant (LA), and anti–b2-glycoprotein I antibodies (anti-b2GPI) which have generally been associated with an increased risk of venous and arterial thrombosis in approximately one-third of SLE patients [6].

Dural sinus thrombosis occurring concomitantly with intracerebral hemorrhage is extremely rare in NPSLE. This case poses an intriguing problem: concomitant dural sinus thrombosis and intracranial bleeding. Even though intracerebral hemorrhage should be considered to be caused by dural sinus thrombosis in this case, treatment with anticoagulation in this situation is still controversial for it may increase the risk of rebleed and deteriorate existing cerebral hemorrhage. We considered noninflammatory microangiopathy and thrombosis were the predominant changes in this patient and cerebral hemorrhage was secondary pathologic change of increased vascular pressure mainly due to venous thrombosis rather than increased vascular wall permeability with inflammation. The patient had a remarkable clinical response to the treatment of anticoagulation and cerebral hemorrhage in brain MRI is dramatically improved after only week of anticoagulation (Figure 3), which demonstrate anticoagulation would not increase the chance of a rebleed in this case.

Hemorrhage can be the initial presentation of dural sinus thrombosis which makes its diagnosis difficult. In this patient, suspicious thrombosis of sagittal sinus and right transverse sinus was suggested by brain MRI (Figure 3) one week after cerebral hemorrhage. In this situation, cerebral vein thrombosis was easily misdiagnosed and mismanaged when cerebral hemorrhage occurred first and it was mentioned in some case reports that Mannitol was administered to reduce intracranial pressure caused by hemorrhage which aggravates thrombosis. Therefore, cerebral sinus venous thrombosis should be included in differential diagnosis if the patient presents with unexplained CNS disorders of sudden onset in complicated NPSLE with thrombophilia.

## References

[1] Jennekens F. G. I., Kater L. (2002). The central nervous system in systemic lupus erythematosus. Part 1. Clinical syndromes: a literature investigation. *Rheumatology*.

[2] Hanly J. G. (2005). Neuropsychiatric lupus. *Rheumatic Disease Clinics of North America*.

[3] (1999). The American College of Rheumatology nomenclature and case definitions for neuropsychiatric lupus syndromes. *Arthritis & Rheumatology*.

[4] Kitagawa Y., Gotoh F., Koto A., Okayasu H. (1990). Stroke in systemic lupus erythematosus. *Stroke*.

[5] Johnson R. T., Richardson E. P. (1968). The neurological manifestations of systemic lupus erythematosus: a clinical-pathological study of 24 cases and review of the literature. *Medicine*.

[6] Love P. E., Santoro S. A. (1990). Antiphospholipid antibodies: anticardiolipin and lupus anticoagulant in systemic lupus erythematosus (SLE) and non-SLE disorders. *Annals of Internal Medicine*.

